# Real-world management and outcomes of older patients with locally advanced esophageal squamous cell carcinoma: a multicenter retrospective study

**DOI:** 10.1186/s12885-023-10710-y

**Published:** 2023-03-28

**Authors:** Yuki Saito, Yasuo Hamamoto, Kenro Hirata, Makoto Yamasaki, Masaya Watanabe, Tetsuya Abe, Yasuhiro Tsubosa, Yoichi Hamai, Kentaro Murakami, Takeo Bamba, Takako Yoshii, Masahiro Tsuda, Masayuki Watanabe, Masaki Ueno, Yuko Kitagawa

**Affiliations:** 1 Division of Gastroenterology and Hepatology, Department of Internal Medicine, Keio University School of Medicine, Tokyo, Japan; 2 Keio Cancer Center, Keio University School of Medicine, 35 Shinanomachi, Shinjuku-Ku, Tokyo, 160-8582 Japan; 3 Department of Gastroenterological Surgery, Graduate School of Medicine, Osaka University, Osaka, Japan; 4 Department of Gastroenterological Surgery, Shizuoka General Hospital, Shizuoka, Japan; 5 Department of Gastroenterological Surgery, Aichi Cancer Center Hospital, Nagoya, Japan; 6 Division of Esophageal Surgery, Shizuoka Cancer Center Hospital, Shizuoka, Japan; 7 Department of Surgical Oncology, Hiroshima University, Hiroshima, Japan; 8 Department of Frontier Surgery, Graduate School of Medicine, Chiba University, Chiba, Japan; 9 Department of Digestive Surgery, Niigata Cancer Center Hospital, Niigata, Japan; 10 Department of Gastroenterology, Saitama Cancer Center, Saitama, Japan; 11 Department of Gastroenterological Oncology, Hyogo Cancer Center, Akashi, Japan; 12 Department of Gastroenterological Surgery, Cancer Institute Hospital of Japanese Foundation for Cancer Research, Tokyo, Japan; 13 Department of Gastroenterological Surgery, Toranomon Hospital, Tokyo, Japan; 14 Department of Surgery, Keio University School of Medicine, Tokyo, Japan

**Keywords:** Esophageal squamous cell carcinoma, Older, Real-world, Treatment, Survival, Esophagectomy, Neoadjuvant chemotherapy, Concurrent chemoradiotherapy

## Abstract

**Background:**

Neoadjuvant chemotherapy (NAC) followed by surgery is the standard treatment for locally advanced esophageal squamous cell carcinoma (ESCC). Chemoradiotherapy (CRT) is an alternative treatment approach. However, both treatments are associated with toxicity, and the optimal treatment for older patients with ESCC is unknown. This study aimed to evaluate the treatment strategies and prognosis of older patients with locally advanced ESCC in a real-world setting.

**Methods:**

We retrospectively evaluated 381 older patients (≥ 65 years) with locally advanced ESCC (stage IB/II/III, excluding T4) who received anticancer therapy at 22 medical centers in Japan. Based on age, performance status (PS), and organ function, the patients were classified into two groups: clinical trial eligible and ineligible groups. Patients aged ≤ 75 years with adequate organ function and a PS of 0–1 were categorized into the eligible group. We compared the treatments and prognoses between the two groups.

**Results:**

The ineligible group had significantly shorter overall survival (OS) than the eligible group (hazard ratio [HR] for death, 1.65; 95% confidence interval [CI], 1.22–2.25; *P* = 0.001). The proportion of patients receiving NAC followed by surgery was significantly higher in the eligible group than in the ineligible group (*P* = 1.07 × 10^–11^), whereas the proportion of patients receiving CRT was higher in the ineligible group than in the eligible group (*P* = 3.09 × 10^–3^). Patients receiving NAC followed by surgery in the ineligible group had comparable OS to those receiving the same treatment in the eligible group (HR, 1.02; 95% CI, 0.57–1.82; *P* = 0.939). In contrast, patients receiving CRT in the ineligible group had significantly shorter OS than those receiving CRT in the eligible group (HR, 1.85; 95% CI, 1.02–3.37; *P* = 0.044). In the ineligible group, patients receiving radiation alone had comparable OS to those receiving CRT (HR, 1.13; 95% CI, 0.58–2.22; *P* = 0.717).

**Conclusions:**

NAC followed by surgery is justified for select older patients who can tolerate radical treatment, even if they are old or vulnerable to enrollment in clinical trials. CRT did not provide survival benefits over radiation alone in patients ineligible for clinical trials, suggesting the need to develop less-toxic CRT.

## Background

Esophageal squamous cell carcinoma (ESCC) is a malignancy with poor prognosis that requires extensive treatment. In Japan, ESCC is most common among the individuals in their 60 s and 70 s. Moreover, 35.7 and 9.8% of patients with ESCC are diagnosed in their 70 s and from early 80 s to late 90 s, respectively [1]; therefore, ESCC is considered a disease of the older people. However, most clinical trials include patients aged 75 years or younger with sufficient organ function and good Eastern Cooperative Oncology Group (ECOG) performance status (PS) [2–4]. Thus, clinical trial-eligible older patients may not represent real-world older patients with respect to toxicity, tolerance, and outcome of cancer treatment.

The standard curative treatment of locally advanced ESCC (clinical stage IB/II/III, excluding T4) is neoadjuvant chemotherapy (NAC) followed by surgery [4–6]. Definitive chemoradiotherapy (CRT) is also a treatment option for patients who cannot undergo surgery for medical reasons or who refuse it [7]. Although treatment regimens and protocols varied among studies, several randomized control trials have shown almost equivalent outcomes associated with both treatment strategies [8–10]. Both treatment strategies are associated with toxicity [3, 11], high mortality, and increased complication rates in older patients [12–14]. Therefore, it is unclear whether NAC followed by surgery or definitive CRT is well tolerated and suitable for older patients in a clinical setting [15]. This study aimed to evaluate the treatment strategies and clinical outcomes in older patients with locally advanced ESCC in a real-world setting.

## Methods

### Patients

A retrospective medical chart review was conducted for older patients with locally advanced ESCC who received anticancer therapy between January 2012 and December 2012 at 22 medical centers (including 10 university hospitals and 10 cancer-specific hospitals), participating institutions of the Japanese Esophageal Oncology Group. This particular period was chosen because it allowed us to assess the longest post-treatment survival since the current treatment strategy, i.e., NAC followed by surgery, became the standard treatment in 2011 [3]. The inclusion criteria were as follows: 1) histologically confirmed thoracic ESCC; 2) age, ≥ 65 years; 3) receiving any anticancer therapy; and 4) stage IB/II/III (excluding T4) based on the 7th UICC-TNM classification. This study was approved by the institutional ethics committee of Keio University Hospital. This study was conducted in accordance with the principles of the Declaration of Helsinki.

### Treatment

Treatments were retrospectively divided into five categories: NAC followed by surgery, surgery without NAC, CRT, radiation, and chemotherapy. The NAC regimens were cisplatin and fluorouracil (*n* = 142, 67.9%); the standard regimen in Japan [3]; cisplatin, fluorouracil, and docetaxel (*n* = 23, 11.0%) [4, 16]; cisplatin, fluorouracil, and doxorubicin (*n* = 14, 6.7%) [17]; neoadjuvant CRT (*n* = 14, 6.7%) [4]; others (*n* = 10, 4.8%); and unknown (*n* = 6, 2.9%). Surgery without NAC category included patients who underwent esophagectomy alone and those who underwent esophagectomy and adjuvant chemotherapy. The CRT category included patients who experienced relapse after CRT and underwent salvage surgery. The chemotherapy regimens used in CRT were cisplatin and fluorouracil (*n* = 55, 77.5%); nedaplatin and fluorouracil (*n* = 4, 5.6%); fluorouracil (*n* = 2, 2.8%); others (*n* = 8, 11.3%); and unknown (*n* = 2, 2.8%). The chemotherapy category included patients who received NAC but showed disease progression during NAC and their tumors became unresectable.

### Assessments

Data on patient characteristics and clinicopathological factors were retrospectively collected. These included age, sex, ECOG PS, Charlson comorbidity index, blood test results (pretherapeutic white blood cell count, hemoglobin level, platelet count, albumin level, total bilirubin level, and creatinine level), main tumor location, cT category, cN category, cM category, details of surgery if performed (date of surgery, fields of lymph node resection, and achievement of curative resection [R0 surgery]), details of neoadjuvant, adjuvant, and CRT, and radiation treatment if performed (date, regimen, and dose), and prognosis. Before treatment, local investigators determined the clinical stage using esophagogastroduodenoscopy and computed tomography. The final outcome was assessed by local investigators as alive, dead because of ESCC, dead because of non-ESCC causes, or unknown.

### Statistical analysis

All statistical analyses were performed with R4.1.1 software (The R Foundation for Statistical Computing). Categorical and continuous data were compared using Fisher’s exact test and Welch’s t-test, respectively. We defined overall survival (OS) as the time from the initiation of the first treatment. Survival probabilities were estimated using the Kaplan–Meier method and the log-rank test. A Cox proportional hazards regression model was used to calculate hazard ratios (HRs). All *P*-values were based on a two-sided hypothesis, and *P-*values < 0.05 were considered statistically significant.

## Results

### Baseline characteristics

**Fig. 1:**
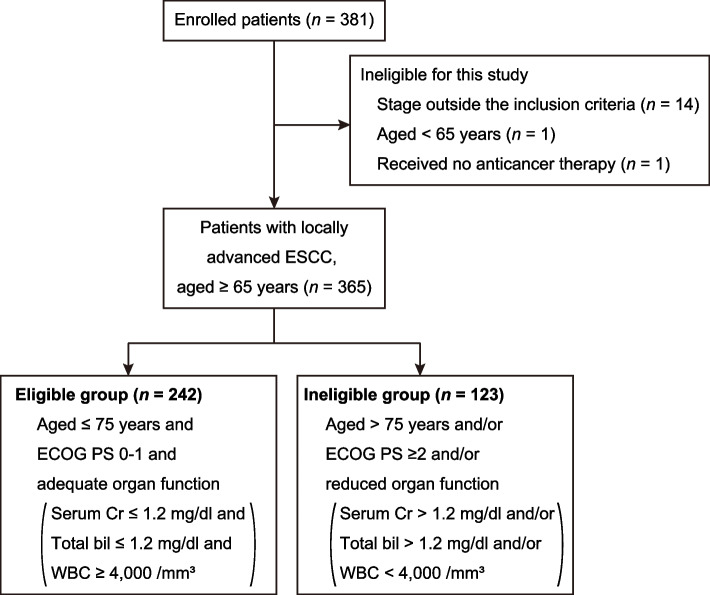
Study consort diagram. ECOG PS, Eastern Cooperative Oncology Group performance status; Cr, creatinine; bil, bilirubin; WBC, white blood cell count

A total of 381 patients with locally advanced ESCC were enrolled in this study. After excluding 16 patients who did not meet the inclusion criteria (stage outside the inclusion criteria [cT1N0M0, *n* = 13; cT4N0M0, *n* = 1], aged < 65 years [*n* = 1], and received no anticancer therapy [*n* = 1]), 365 patients were included in the final analysis (Fig. 1). The baseline clinical characteristics of the patients are summarized in Table 1. Eighty-one patients (22.2%) aged > 75 years, with a median age at diagnosis of 72 years (range, 65–89 years). The ECOG PS was 0, 1, 2, 3, and 4 for 259 (71.0%), 94 (25.8%), 8 (2.2%), 3 (0.8%), and 1 (0.3%) patients, respectively. The Charlson comorbidity index, a validated method for estimating the risk of mortality from comorbid diseases [18], was 0, 1–2, and ≥ 3 for 175 (47.9%), 147 (40.3%), and 43 (11.8%) patients, respectively. A total of 209 patients (57.3%) underwent NAC followed by surgery; 55 (15.1%) underwent surgery without NAC; and 71 (19.5%), 22 (6.0%), and 8 (2.2%) received CRT, radiation, and chemotherapy, respectively.

**Table 1:** Baseline characteristics of the patients

	Eligible group (*n* = 242)	Ineligible group (*n* = 123)	All patients (*n* = 365)	*P*-value
Age ― years	70.1 ± 2.87	76.0 ± 5.12	72.1 ± 4.68	1.8 × 10^–23^
Male sex ― *n* (%)	209 (86)	106 (86)	315 (86)	1.00
ECOG PS ― *n* (%)				7.6 × 10^–8^
0	188 (78)	71 (58)	259 (71)	
1	54 (22)	40 (33)	94 (26)	
2–4	0 (0)	12 (10)	12 (3)	
Charlson comorbidity index ― *n* (%)				9.0 × 10^–5^
Low: 0	134 (55)	41 (33)	175 (48)	
Medium: 1–2	88 (36)	59 (48)	147 (40)	
High: 3–6	20 (8)	23 (19)	43 (12)	
Tumor site ― *n* (%)				0.96
Ut	36 (15)	17 (14)	53 (15)	
Mt	118 (49)	62 (50)	180 (49)	
Lt	88 (36)	44 (36)	132 (36)	
T stage ― *n* (%)				0.60
T1b	26 (11)	10 (8)	36 (10)	
T2	48 (20)	29 (23)	77 (21)	
T3	168 (69)	84 (68)	252 (69)	
N stage ― *n* (%)				0.65
N0	69 (29)	37 (30)	106 (29)	
N1	107 (44)	47 (38)	154 (42)	
N2	52 (22)	29 (24)	81 (22)	
N3	14 (6)	10 (8)	24 (7)	
Clinical stage ― *n* (%)				0.32
IB	24 (10)	17 (14)	41 (11)	
IIA/IIB	85 (35)	35 (29)	120 (33)	
IIIA/IIIB/IIIC	133 (55)	71 (58)	204 (56)	
Treatment				1.9 × 10^–13^
NAC followed by surgery ― *n* (%)	169 (70)	40 (33)	209 (57)	
Curative resection ― *n* (%)	161 (67)	39 (32)	200 (55)	1.00
Surgery ― *n* (%)	27 (11)	28 (23)	55 (15)	
Curative resection ― *n* (%)	25 (10)	26 (21)	51 (14)	1.00
CRT ― *n* (%)	36 (15)	35 (28)	71 (20)	
Radiation dose ― *n* (%)				5.1 × 10^–3^
≥ 50 Gy	36 (15)	28 (23)	64 (18)	
< 50 Gy	0 (0)	4 (3)	4 (1)	
Unknown	0 (0)	3 (2)	3 (1)	
Radiation ― *n* (%)	3 (1)	19 (15)	22 (6)	
Radiation dose ― *n* (%)				0.26
≥ 50 Gy	2 (1)	18 (15)	20 (5)	
< 50 Gy	1 (0)	1 (1)	2 (1)	
Chemotherapy ― *n* (%)	7 (3)	1 (1)	8 (2)	
Blood test				
White blood cell count ― per μL	6,691 ± 1,971	6,337 ± 2,772	6,572 ± 2,275	0.21
Hemoglobin ― g/dL	13.3 ± 1.46	12.5 ± 1.68	13.1 ± 1.59	2.0 × 10^–6^
Platelet count ― × 10^3^ per μL	235 ± 63.3	224 ± 78.9	231 ± 69.0	0.19
Albumin ― mg/dL	4.03 ± 0.48	3.85 ± 0.46	3.97 ± 0.48	3.3 × 10^–4^
Total bilirubin ― mg/dL	0.66 ± 0.27	0.81 ± 0.56	0.71 ± 0.40	5.9 × 10^–3^
Creatinine ― mg/dL	0.82 ± 0.18	0.90 ± 0.33	0.85 ± 0.24	8.9 × 10^–3^

Plus-minus values are means ± SD

*ECOG PS* Eastern Cooperative Oncology Group performance status, *NAC* Neoadjuvant chemotherapy, *CRT* Chemoradiotherapy

In all 365 patients, the median OS was 5.4 years. The median follow-up time for survivors was 5.1 years (range, 0.2–6.7 years). The 1-, 3-, and 5-year OS rates of the entire cohort were 85, 61, and 53%, respectively.

### Comparison between the clinical trial eligible group and ineligible group

**Fig. 2:**
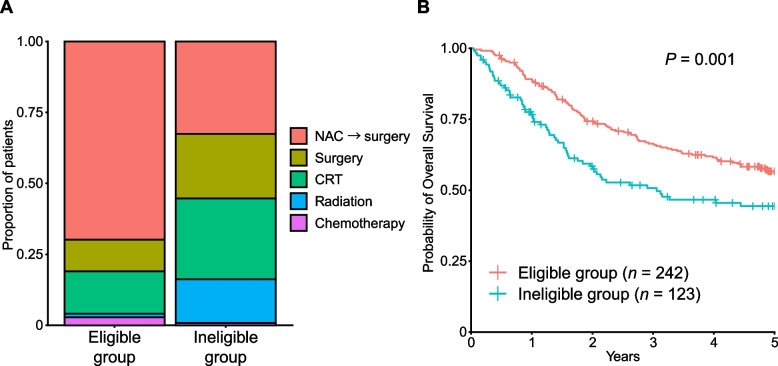
Comparison between eligible and ineligible group. **A** Treatments by groups. **B** Kaplan–Meier curves for overall survival by groups. NAC → surgery, neoadjuvant chemotherapy followed by surgery; CRT, chemoradiotherapy

We classified these patients into two groups (clinical trial eligible and ineligible groups) based on age, ECOG PS, and organ function (Fig. 1 and Table 1). While the eligibility criteria were different between clinical trials, our criteria were constructed based on the eligibility criteria of recent JCOG clinical trials [3, 4]. Patients aged ≤ 75 years with adequate organ function and an ECOG PS 0 or 1 comprised the eligible group. In the eligible group (*n* = 242), 169 (69.8%), 27 (11.2%), 36 (14.9%), 3 (1.2%), and 7 (2.9%) patients received NAC followed by surgery, surgery without NAC, CRT, radiation, and chemotherapy, respectively (Fig. 2A). In the ineligible group (*n* = 123), 40 (32.5%), 28 (22.8%), 35 (28.5%), 19 (15.4%), and 1 (0.8%) patients received NAC followed by surgery, surgery without NAC, CRT, radiation, and chemotherapy, respectively (Fig. 2A). As expected, the proportion of patients who received NAC followed by surgery was significantly higher in the eligible group than in the ineligible group (*P* = 1.07 × 10^–11^), whereas the proportion of patients receiving CRT and surgery without NAC was higher in the ineligible group (*P* = 3.09 × 10^–3^ and 5.02 × 10^–3^, respectively). The ineligible group had significantly shorter OS than the eligible group (HR for death, 1.65; 95% confidence interval [CI], 1.22–2.25; *P* = 0.001) (Fig. 2B). Our results suggest that the majority of patients in the ineligible group received less-toxic treatments given that they were not tolerable to NAC followed by surgery.

### Different prognosis by treatment in each group

**Fig. 3:**
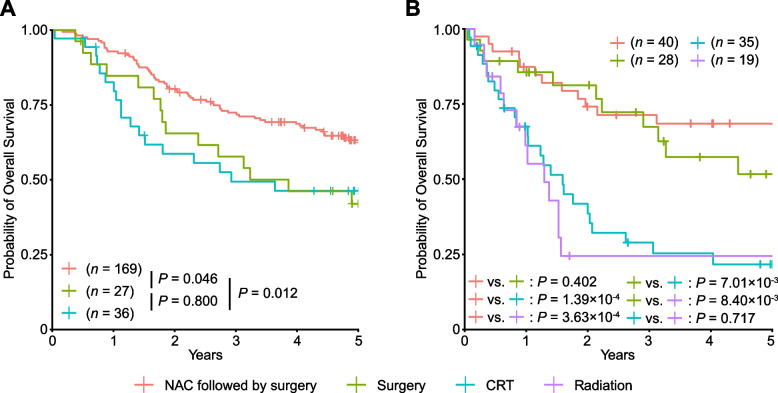
Kaplan–Meier curves for overall survival by treatment. Kaplan–Meier curves for overall survival by treatment in the eligible group (**A**) and ineligible group (**B**). Patients receiving radiation in the eligible group (*n* = 3) and those receiving chemotherapy in the eligible group (*n* = 7) and ineligible group (*n* = 1) were not evaluated because of the small number of samples

We next evaluated the prognosis by treatment in the clinical trial-eligible and ineligible groups. In the eligible group, the OS of patients who received NAC followed by surgery was significantly longer than those who received surgery without NAC (HR, 0.56; 95% CI, 0.32–0.99; *P* = 0.046) or those who received CRT (HR, 0.52; 95% CI, 0.31–0.86; *P* = 0.012; Fig. 3A). This is consistent with previous clinical trial [3], confirming that NAC followed by surgery is the recommended therapy for patients eligible for clinical trials. The OS of patients who underwent surgery without NAC was almost comparable to that of patients receiving CRT (HR, 0.92; 95% CI, 0.47–1.80; *P* = 0.800), which may indicate that CRT is a good treatment option for those who are not candidates for NAC followed by surgery.

**Fig. 4:**
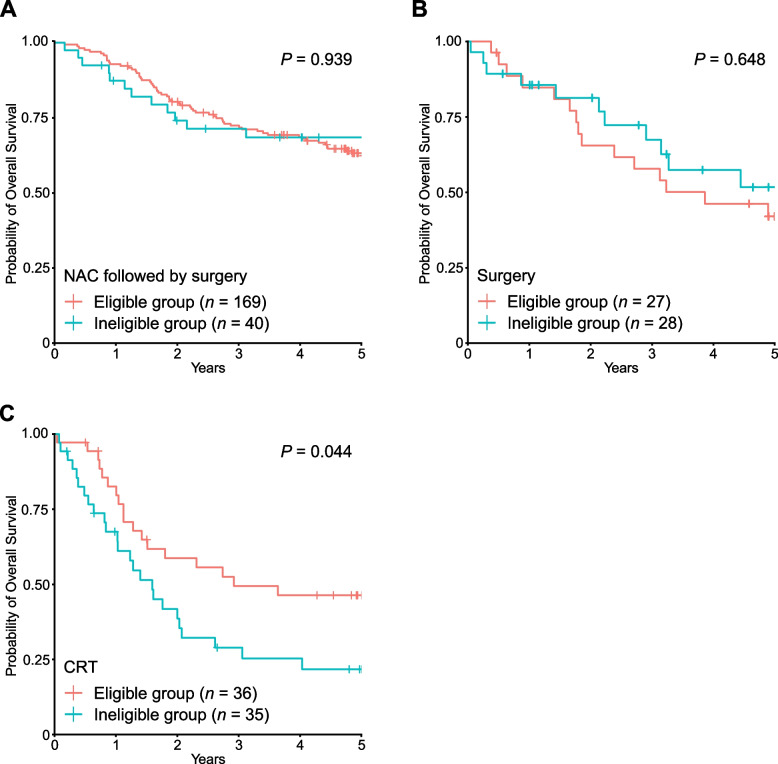
Kaplan–Meier curves for overall survival by group in patients receiving individual treatment. Kaplan–Meier curves for overall survival by group in patients receiving NAC followed by surgery (**A**), surgery without NAC (**B**), and CRT (**C**)

In the ineligible group, patients receiving any surgical treatment (NAC followed by surgery and surgery without NAC) had significantly longer survival than those receiving radiation or CRT (HR, 0.31; 95% CI, 0.19–0.51; *P* = 5.35 × 10^–6^; Fig. 3B). When evaluating those receiving NAC followed by surgery in the whole cohort, the OS of the ineligible group was not significantly different from that of the eligible group (HR, 1.02; 95% CI, 0.57–1.82; *P* = 0.939; Fig. 4A). Similarly, for patients who underwent surgery without NAC, there was no significant difference in OS between the two groups (HR, 0.84; 95% CI, 0.39–1.79; *P* = 0.648; Fig. 4B). Taken together, our results suggest that surgical treatment is the recommended therapy for select older patients with ESCC.

Another interesting observation was the comparable OS of patients receiving CRT and radiation in the ineligible group (HR of death for those receiving radiation compared with those receiving CRT, 1.13; 95% CI, 0.58–2.22; *P* = 0.717; Fig. 3B). This result suggests that the addition of chemotherapy does not provide a prognostic advantage for patients in the ineligible group. Although data on toxicity and treatment completion rates were not available in this cohort, this may be due to the lower completion rate of CRT in the ineligible group. In fact, all 36 patients in the eligible group received ≥ 50 Gy, but 4 patients (11.4%) in the ineligible group received < 50 Gy (Table 1), probably because of early termination of CRT in the ineligible group. Consistent with this hypothesis, the OS of patients receiving CRT in the ineligible group was significantly shorter than that of those receiving CRT in the eligible group (HR, 1.85; 95% CI, 1.02–3.37; *P* = 0.044; Fig. 4C). The development of less toxic CRT optimized for older patients may be necessary to improve their survival.

### PS and comorbidity affect the treatment strategy and prognosis

**Fig. 5:**
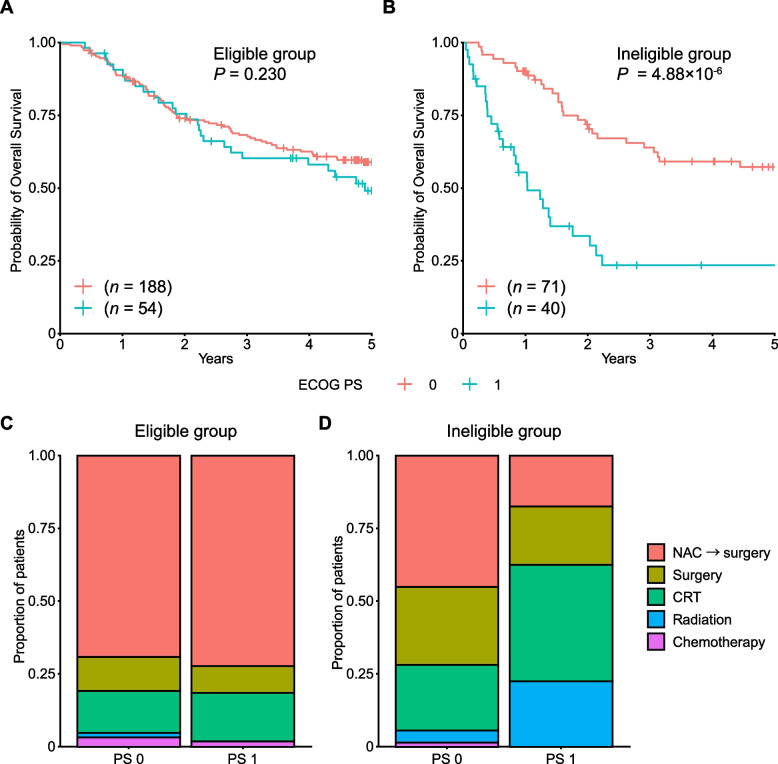
Comparison by PS in each group. Kaplan–Meier curves for overall survival by PS in the eligible group (**A**) and ineligible group (**B**). Treatment by PS in the eligible group (**C**) and ineligible group (**D**). Patients with PS ranging from 2 to 4 in the ineligible group were not evaluated because of the small number of samples (*n* = 12)

Finally, we evaluated the prognostic effect of ECOG PS on survival in individual groups. In the eligible group, patients with PS 1 showed almost comparable OS to those with PS 0 (HR, 1.30; 95% CI, 0.85–2.01; *P* = 0.230; Fig. 5A). In contrast, in the ineligible group, patients with PS 1 showed a significantly worse prognosis than those with PS 0 (HR, 3.45; 95% CI, 2.03–5.86; *P* = 4.88 × 10^–6^; Fig. 5B). It is likely the result of the patients with PS 1 in the ineligible group receiving less intensive treatment. In the ineligible group, patients with PS 1 received considerably more radiation than did patients with PS 0, whereas those with PS 1 received significantly less NAC followed by surgery (*P* = 7.79 × 10^–3^ and 3.80 × 10^–3^, respectively; Fig. 5C and D).

**Fig. 6:**
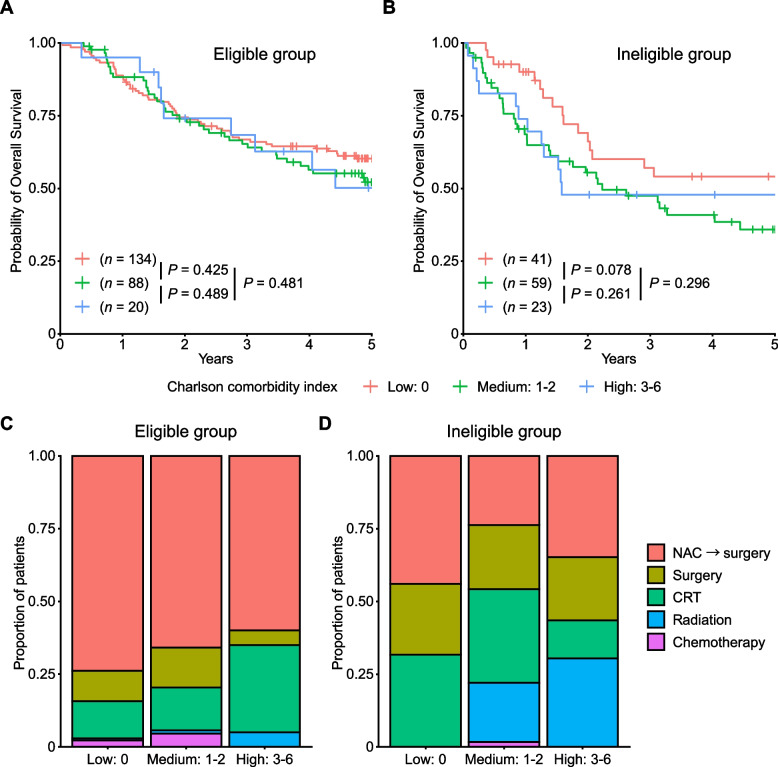
Comparison by Charlson comorbidity index in each group. Kaplan–Meier curves for overall survival according to the Charlson comorbidity index in the eligible group (**A**) and ineligible group (**B**). Treatment by PS in the eligible group (**C**) and ineligible group (**D**)

We also evaluated the prognostic effect of the Charlson comorbidity index. Patients with low, medium, and high scores showed almost comparable OS in the eligible group (Fig. 6A). In the ineligible group, patients with medium and high scores showed a slight but non-significant inferior OS than those with low scores in the ineligible group (HR for patients with medium and high scores compared with those with low scores, 1.61; 95% CI, 0.93–2.78; *P* = 0.086; Fig. 6B). In the eligible group, treatment was not significantly different between patients with low, medium, and high scores. In the ineligible group, patients receiving radiation were significantly different (*P* = 2.97 × 10^–4^); 20% and 30% of patients with medium and high scores received radiation alone, respectively, whereas none of the patients with low scores received the same treatment (Fig. 6C and D).

## Discussion

In this retrospective, multicenter study, we investigated real-world clinical practice for older patients with locally advanced ESCC. We classified the older patients into clinical trial-eligible and ineligible groups, where distinct prognoses and courses of treatment were considered. In the ineligible group, PS and comorbidities affected the treatment strategy and prognosis (Figs. 5 and 6). Our results demonstrate the heterogeneous nature of older patients, ranging from fit patients feasible to aggressive treatment to frail patients not tolerable to such treatments.

One important finding in our study was that OS was comparable between the eligible and ineligible groups after surgical resection (Fig. 4A and B). Esophagectomy for older patients is controversial; several studies reported the satisfactory prognosis of esophagectomy in older patients without any increased morbidity or mortality [19–21], whereas other studies reported increased postoperative mortality risk and reduced survival of older patients undergoing esophagectomy in comparison with young patients [13, 14, 22]. In our retrospective study, among the patients who underwent esophagectomy, older patients who did not meet the clinical trial inclusion criteria showed an OS comparable to those who met the criteria. Our results suggest that surgery (esophagectomy) should be considered for select older patients with ESCC, even if they are old or vulnerable to enrollment in clinical trials.

Meanwhile, patients receiving CRT in the ineligible group showed worse prognosis than those in the eligible group (Fig. 4C). In the ineligible group, patients receiving CRT showed an OS comparable to those receiving radiation alone (Fig. 3B). This is consistent with a recent retrospective analysis showing no significant difference in survival between CRT (with cisplatin and fluorouracil) and radiation alone among older patients with ESCC [23]. In fact, intolerance to CRT toxicity has been reported in older patients [12, 24], and dose adjustment and discontinuation are required for many patients. Our results, together with those of previous reports, suggest the necessity of developing less-toxic CRT suitable and feasible for older patients. Recently, a randomized phase 3 clinical trial conducted in China reported an improved 2-year OS of CRT with S-1 compared with radiation alone in older patients (aged 70–85 years) with ESCC [25]. Although this may be an attractive regimen, the enrolled patients were relatively young and had a good ECOG PS and low Charlson comorbidity index. The enrolled population may not be vulnerable [26], and many of them appear to be classified into eligible groups according to our criteria. Therefore, further clinical trials are needed to determine the optimal treatment for frail older patients [27].

Our study had several limitations. First, this study collected limited information about patient characteristics, and we did not evaluate other factors such as preoperative pulmonary function. Second, this study lacked comprehensive geriatric assessment. Finally, because this was a retrospective study, the treatment was heterogeneous. We classified the treatment into five categories, but the treatment was heterogeneous even in individual categories with respect to the regimen and dose of treatment. Nevertheless, these heterogeneities reflect real-world management of older patients with ESCC.

## Conclusions

Our retrospective, multicenter study suggests that NAC followed by surgery is justified for select older patients who can tolerate radical treatment, even if they are old or vulnerable to enrollment in clinical trials. In addition, CRT did not provide a survival benefit over radiation alone in patients ineligible for clinical trials, suggesting the need to develop less-toxic CRT. Prospective studies with large sample sizes are recommended to determine the optimal treatment for older patients with ESCC.

## Data Availability

The dataset generated and/or analysed during the current study is not publicly available due to limitations of ethical approval involving the patient data and anonymity but is available from the corresponding author on reasonable request.

## Abbreviations

ESCC: Esophageal squamous cell carcinoma
NAC: Neoadjuvant chemotherapy
CRT: Chemoradiotherapy
ECOG: Eastern Cooperative Oncology Group
PS: Performance status
OS: Overall survival
HR: Hazard ratio
CI: Confidence interval
